# An overview of molecular epidemiology of hepatitis B virus (HBV) in India

**DOI:** 10.1186/1743-422X-5-156

**Published:** 2008-12-19

**Authors:** Sibnarayan Datta

**Affiliations:** 1 ICMR Virus Unit Kolkata, Infectious Diseases & Beleghata General Hospital Campus, 57 Dr. Suresh Chandra Banerjee Road, Kolkata 700010, India

## Abstract

Hepatitis B virus (HBV) is one of the major global public health problems. In India, HBsAg prevalence among general population ranges from 2% to 8%, placing India in intermediate HBV endemicity zone and the number of HBV carriers is estimated to be 50 million, forming the second largest global pool of chronic HBV infections. India is a vast country, comprised of multiracial communities with wide variations in ethnicity and cultural patterns, which is attributable to its geographical location, gene influx due to invasion and/or anthropological migrations in the past. Moreover, recent increase in trade, trafficking and use of illicit drugs has also considerably influenced the epidemiology of HBV, specifically in the eastern and north eastern parts of India. However, data on the molecular epidemiology of HBV in India is scanty. HBV genotypes A and D have been well documented from different parts of mainland India. Interestingly, in addition to genotypes A and D, genotype C having high nucleotide similarity with south East Asian subgenotype Cs/C1 strain, have been detected exclusively from eastern Indian HBV carriers, suggesting a recent introduction. Thus, compared to other parts of India, the molecular epidemiology of HBV is naturally distinct in eastern India. Very recently, taking the advantage of circulation of three distinct HBV genotypes within the population of eastern India, different aspects of HBV molecular epidemiology was studied that revealed very interesting results. In this study, the clinical significance of HBV genotypes, core promoter and precore mutations, possible routes of introduction of HBV genotype C in eastern India, the clinical implications of *x *gene variability, prevalence of the AFB_1 _induced p53 gene codon 249 mutation, the transmission potentiality of HBV among asymptomatic/inactive or occult HBV carriers and the genetic variability of HBV persisting in the PBL was investigated. In this manuscript, the information available on the molecular epidemiology of HBV in India has been reviewed and the results of studies among the eastern Indian population have been summarised.

## Background

Hepatitis B virus (HBV) is one of the major global public health problems. HBV infection is the 10^th ^leading cause of death and HBV related hepatocellular carcinoma (HCC) is the 5^th ^most frequent cancer worldwide. About 30 percent of the world's population has serological evidence of current or past infection with HBV. Of these, an estimated 350 million are chronically infected with HBV and approximately 1 million persons die annually from HBV-related chronic liver diseases, including severe complications such as liver cirrhosis (LC) and HCC [1].

HBV is distributed worldwide, but its prevalence varies significantly between different populations of the world. Based on the prevalence of HBV surface antigen (HBsAg) carrier rate in the general population, sub-Saharan African, East Asian and Alaskan populations are classified as having high HBV endemicity (HBsAg carriage > 8%), while the populations of southern parts of Eastern and Central Europe, the Amazon basin, the Middle East, and the Indian subcontinent are classified as intermediate HBV endemicity (HBsAg carriage 2–7%), and the populations in western and northern Europe, North America, and Australia are classified as low HBV endemic (HBsAg carriage < 2%) regions [2].

## The HBV genome and origin of genetic diversity

HBV belongs to the virus family *Hepadnaviridae *(infecting different avian and mammalian hosts), which includes several genera of partially double stranded DNA genome of approximately 3.2 kb length, generated through reverse transcription from a longer intermediate RNA (approximately 3.5 kb, generally referred to as pregenomic RNA or pgRNA) [3]. The HBV genome encodes four partially overlapped open reading frames (ORF): the *surface (preS1, preS2, S)*, *core (precore, core)*, *polymerase *and the '*x' *genes respectively. High genetic variability is a characteristic feature of the HBV as the viral polymerase lacks proofreading activity and uses an RNA intermediate during its replication [3,4]. On the other hand, the extreme overlapping of the open reading frames of the HBV genome limits the possibility of fixation of all these mutations [5]. These opposite aspects render the substitution rate of HBV to an intermediate level between RNA and DNA viruses [5,6].

Such a replication system makes random errors during genomic replication, which are the source of genetic variation, upon which natural selection can act, leading to evolution of the HBV genome [7]. The nucleotide substitution rate, for HBV has been estimated to be 1.4 – 5.0 × 10^-5 ^per site per year, being 10 fold superior than other DNA viruses, but the rate of synonymous (silent) substitutions is higher than the rate of non-synonymous substitutions, suggestive of a constrained evolution of the HBV genome [5,8,9]. In contrast, in a liver transplantation setting, the mutation rate has been found to be almost 100-fold higher [10], while mutation rate is negligible in silent or occult HBV infection, where there is minimal host response over many decades [11]. However, Hannoun et al., [12] calculated a mean frequency of fixation of nucleotide substitution of a wider range (2.1–25 × 10^-5 ^nucleotide change per site per year) depending on the HBeAg/anti-HBeAg status of the host. Thus it appears that, host-virus interaction and immune selective pressures, imposed by the host immune system, either naturally or medically, can affect the variability of the HBV genome.

Random errors/variations in the HBV genome, occurring due to long periods of persistence and immune selection pressures operating at the population level have led to the emergence of distinct genotypes and their subgenotypes in specific geo-ethnic populations, and being transmission competent these variants stably circulate within the given geo-ethnic population [6,13-15]. In addition, certain mutations may also emerge under medical pressures (vaccine, or antiviral therapy), which are selected at the individual level. During specific phases of chronic HBV infection, mutations (e.g. 587^A^, 1896^A^, 1762^T^/1764^A ^etc.) emerge that are advantageous for escaping the natural or therapy induced antiviral immune pressure and thus favours viral persistence.

## HBV genetic diversity: genotypes & subgenotypes

Classically, HBV strains were distinguished by the presence of two pairs of mutually exclusive serotype determinants '*d*'/'*y*' and '*w*'/'*r*', in the HBsAg along with the main antigenic determinant 'a', which led to the description of 4 serotypes, namely *adw, adr, ayw *or *ayr*. Additional serotypes were subsequently characterized leading to the description of nine serotypes namely *ayw1, ayw2, ayw3, ayw4, ayr, adw2, adw4, adrq+ *and *adrq*- and a distinct geographical pattern for the distribution of serotypes was also documented [6]. However, with the advent of molecular biological techniques and advanced computational methods for the phylogenetic analysis of complete viral genome sequences, HBV genotypes and subgenotypes have been described, that have largely replaced the classical serotype based classification of HBV strains.

**Figure 1:**
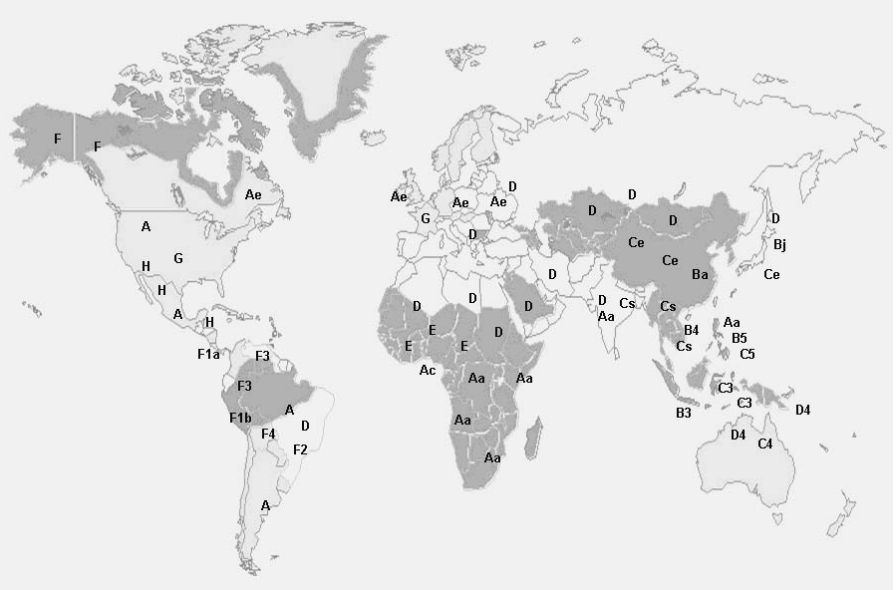
**Worldwide distribution patterns of HBV genotypes and subgenotypes**. Regions with high, intermediate and low endemicity are shown by grey, light grey and white shades respectively.

Based on more than 8% genetic variability among HBV strains found worldwide, eight HBV genotypes namely A, B, C, D, E, F, G, and H have been well established [16-19]. Further extensive phylogenetic analyses of the HBV genotypes have resulted in recognition of subgenotypes of genotypes A, B, C, D and F, based on more than 4% intra-genotypic divergence. Until now, the presence of 5 subgenotypes have been recognized for each of the HBV genotypes A, B, C and D, while 4 subgenotypes have been well reported for genotype F [15]. Having evolved distinctly in specific geo-ethnic populations, HBV genotypes/subgenotypes have a distinct geographical distribution pattern (Figure 1).

## Clinical significance of HBV genetic variability

Accumulating evidences clearly indicate that HBV genotypes/subgenotypes can significantly influence HBeAg seroconversion rates, viremia levels, mutational patterns that could significantly influence the heterogeneity in clinical manifestations and even response to antiviral therapy [13,14,20]. More fascinatingly, the emergence of the most widely studied clinically important mutations (e.g. 1896^A^, 1762^T^/1764^A ^etc.) that are significantly associated with HBV e antigen (HBeAg) negative chronic infections have been shown to be subjective to the infecting HBV genotypes. The precore (PC 1896^A^) mutation creates a premature stop codon at position 28 precluding the HBeAg expression and is specifically frequent among patients infected with genotypes B, C and D [14]. The basal core promoter (BCP) double mutations 1762^T^/1764^A ^downregulate HBeAg production and are associated with chronic HBV infection leading to HCC [21], occur frequently among patients infected with HBV genotypes A, C and F [14]. Recent studies have also shown that certain HBV genotypes may also influence the development of HBV vaccine escape mutants and therefore the efficacy of HBV vaccination is dependent on the HBV genotypes of the given population [22]. Due to this nexus between HBV genotypes and the known clinically important mutations, certain genotypes appear to be significantly associated with more severe consequences, than others.

Owing to the distinct geographic distribution patterns of HBV genotypes, only one or two HBV genotypes have been reported to circulate in most of the populations studied so far. Thus most comparative studies on clinical significance of HBV genotypes among a population of similar ethnicity have remained restricted mainly to two distinct genotypes (genotype B versus genotype C in East Asian countries and genotype A versus genotype D in Europe and India) [14,23]. The results of these studies have demonstrated marked differences in the virological, epidemiological and clinical characteristics among the compared genotypes. Thus molecular epidemiological studies in geographical regions where more than two HBV genotypes are circulating in the same population may reveal more interesting aspects of the HBV genetic variability.

Interestingly, in addition to diverse clinical manifestations between different HBV genotypes, it has been noted that the predominant mode of transmission varies significantly between populations. Vertical or perinatal transmission is predominant in HBV endemic East Asian countries where HBV genotypes B and C are prevalent, whereas horizontal transmission is the main route of infection in Africa, Europe, Middle East and Indian subcontinent, where genotypes A and D prevail [14]. This is more prominent in case of genotype G, as most genotype G isolates have been isolated from homosexual men and confined to the USA and Europe [24,25]. This raises an important question about the genotype restricted patterns of HBV compartmentalization and thus distinct modes of transmission of HBV, which have important implications in the molecular epidemiology of HBV.

Due to their characteristic geo-ethnic distribution patterns, HBV genotypes/subgenotypes has been successfully used to correlate the population migration with shifting epidemiology and introduction of new HBV strains in a given region. In countries with a history of human migration, the prevalence of different HBV genotypes have been shown to reflect the original HBV genotype distribution among the immigrants [6,14]. Apart from human migration, certain high-risk behavioural patterns, such as intravenous drug abuse, have been reported to rapidly influence the molecular epidemiology of HBV genotypes/subgenotypes in a given region [26,27]. Thus the investigation and surveillance of HBV genotypes and subgenotypes, using molecular epidemiological techniques help in tracing the routes of influx of newer strains and are thus important for designing effective preventive strategies.

## Aetiology of HBV related HCC

HCC is one of the most malignant cancers, increasing by estimated 5, 60,000 new cases per year, and the third among most common cause of death among men [28]. The main causes of HCC are chronic infection with HBV, long term dietary exposure to aflatoxin B_1 _(AFB_1_), chronic alcoholism, besides other causes [29]. Worldwide, the highest incidences of HCC and the youngest patients with this tumour are found in China, Taiwan, and sub-Saharan Africa, each of which is hyperendemic for HBV infection with either HBV genotypes B, C (in China, Taiwan) or genotype A (in sub Saharan Africa) and a high rate of dietary exposure to the fungal toxin, AFB_1_[14,29].

From a large number of molecular epidemiological studies, persuasive evidence has now accumulated that in HCC endemic regions, AFB_1 _and HBV interact synergistically in the aetiology and pathogenesis of HBV related HCC [29-31]. Several groups have shown that one of the gene products of HBV, the HBx binds to and inactivates the p53 protein [32,33]. It has been experimentally demonstrated that besides physically interacting with p53, HBx may induce inactivating mutations in the p53 gene either by down regulating the detoxification of AFB_1 _[34], or simply by increasing the transversion frequency [35], resulting in a specific guanine to thymine transversion mutation in the third nucleotide position of codon 249 (AGG to AGT, leading to an arginine-to-serine substitution) in the p53 protein [36]. This mutation is considered as a reliable biomarker for the development of HCC in geographical regions where the chronic exposure to HBV and dietary AFB_1 _are very high.

Experimental evidences have established that HBx is a multifunctional protein with oncogenic potentials, and is capable of interacting and modulating the normal function of a large battery of cellular factors, leading to deregulation of the normal cell activities, leading to HCC [37]. However considering the high incidence of HBV genotypes A, B or C in the high HCC incidence zones of the world, it seems that HBx encoded by certain HBV genotypes have higher hepatocarcinogenic potentials than HBx of other genotypes. Moreover, emergence of certain HBV genotype associated mutations (K130M, V131I) in the *x *ORF has also been shown to predict the development of HCC [21]. Despite its importance in HCC development, the clinical significance of the genetic variability of the *x *genetic region still remains poorly understood [38]. Hence, molecular epidemiological studies targeting HBx genetic variability and occurrence of AFB1 induced p53 mutations may be helpful in assessment and surveillance of HCC risk in regions where chronic HBV and AFB_1 _exposure is high.

## Epidemiology of HBV infections in India

Infectious diseases are a major cause of deaths in South Asia, including India. HIV, Tuberculosis and chronic hepatitis B continue to threaten the lives of millions in India. India now has the second largest population with AIDS and HIV infection in the world [39], signifying the rapid change in the epidemiology of parenterally/sexually transmitted viral infections via different modes [40,41]. High rates of these infections in many South Asian countries are attributable to poverty, unhygienic living conditions, illiteracy, unsafe blood supply, poor medical facilities, and reuse of contaminated syringes, unsafe sexual practice, and frequent use of intravenous drugs.

According to the WHO report on prevention of HBV in India [42], HBsAg prevalence among general population ranges from 0.1% to 11.7%, being between 2% to 8% in most studies. HBsAg prevalence rate among blood donors ranged from 1% to 4.7%. With the exception of higher HBsAg positivity in some North Eastern states (~7%), no substantial geographical variation was apparent in other parts of India. Considering, on an average, HBsAg carrier rate of 5%, the total number of HBV carriers in the country was estimated to be about 50 million that forms nearly 15% of the entire pool of HBV carriers in the world and is the second largest pool of chronic HBV infections in the world [42]. Using conservative prevalence estimates of different HBV seromarkers for estimating the number of HBV infections and serious disease outcomes in population, it was predicted that over 9 million are estimated to acquire HBV infection during their lifetime, an estimated 1,507,000 will develop chronic HBV infection, and nearly 200,000 will die of acute or chronic consequences of HBV infection [42], which clearly indicates an impending danger.

Recently, in contrast to the mainland India, very high rates of HBsAg have been recorded among certain primitive tribes of Andaman and Nicobar Islands. Studies showed hyperendemic HBV infection, with HBsAg carrier rates ranging from 23.3% among the Nicobarese tribe, 37.8% among the Shompen tribe, [43], 11.6% among the Karen [44], and over 65% among the Jarawa tribe [45]. The HBsAg prevalence rates among the Jarawa are the highest ever reported in the world.

India is a vast country, comprised of multiracial communities with wide variations in culture, ethnicity, food habits, lifestyle of different communities and thus infectious and chronic disease patterns [46]. Geographical location of India is between West and Central Asian countries and East Asian countries, having different HBV genotype distributions. Gene flow from these neighbouring countries, due to anthropological migration in the past has contributed to considerable genetic, geographic and socio-cultural diversity of the Indian population [47,48]. In addition, more than 200 years of colonial rule in India have been suggested to have important epidemiological implications on the genotypic distribution of HBV [49]. Genetic studies on mtDNA and Y chromosomal DNA in the Indian population have also attested to the significant European admixture [50]. This multiethnic origin of the Indian populations is also reflected in the HBV genotype distribution in different parts of the country. Moreover, recent increase in trade, trafficking and use of illicit drugs and frequent visits to and from different countries have also considerably influenced the epidemiology of HBV and other parenteral infections in India and specially in the eastern and north eastern parts of India [51-53].

## Modes of HBV transmission in India

A large study involving 8575 pregnant women from Northern India, documented HBsAg carrier rate in antenatal mothers to be 3.7%, HBeAg carrier rate 7.8% and vertical transmission was observed in 18.6% [54]. Taking into account the low percentage of possible vertical transmission, it appears that the potential of perinatal HBV transmission in India is similar to Africa but lower than that in East Asia. It has been estimated that HBV infection is largely acquired by horizontal transmission in childhood and perinatal transmission plays a less important role [54,55]. A study from Eastern India demonstrated that HBsAg prevalence among antenatal mothers attending a maternity home in Calcutta is in conformity with national average of HBsAg prevalence (3–5%) in India [56]. However, both HBeAg positivity (1%) and the level of serum HBV DNA among antiHBe positive cases being low, the infectivity status among the antenatal mothers is assumed to be low, suggesting that perinatal transmission of infection from mother to infants is not an important route of HBV transmission in India [55,56].

The peaking of infection rates in adulthood in Indian population also suggests a close relationship of acquisition of infection in the adults [57]. In an earlier study, frequent exposure to percutaneous injuries, repeated use of parenteral injections for trivial illnesses and the untrained para-medical personnel, lacking in knowledge about modes of sterilization in primary care centres have been found to be the major factors that facilitate transmission of HBV, as well as other viruses in this population [57]. Apart from exposure from extraneous sources, intrafamilial aggregation of HBV infected persons in a family has been well documented in India [55]. HBsAg contamination of surfaces is widespread in homes of chronically infected persons [58], which may explain the non-sexual interpersonal spread of HBV such as among household contacts. Household contacts of subjects with chronic HBV infection are known to be at high risk of acquiring infection through multiple modes [59]. A serological survey on 722 family members of 215 HBV infected Index cases of eastern India revealed that intrafamilial horizontal transmission is more significant mode of transmission than sexual mode of transmission in later life for maintaining HBV carrier pool in this community [55]. In an another study on HBV transmission in the families of 12 chronic liver disease patients from Northern India, horizontal transmission pattern was found in 50%, vertical transmission pattern in 17% and by both patterns in rest of the families on the basis of homology between the viral sequences of the members of same family [60].

Previous studies among the primitive tribes of the Andaman and Nicobar islands have shown high endemicity of HBV infection [43]. Horizontal transmission through close contact with carriers and perinatal routes was identified as an important mode of transmission of HBV in these tribal communities. Besides, use of unsafe injections represents an independent risk factor for acquiring HBV infection in this island population [43]. Very high endemicity of HBV infection in the tribal populations have been suggested to be due to their association with a number of socio-culture practices like endogamy, bloodletting, scarification, and tattooing and eating of orally processed food.

## Distribution of HBV genotypes and subgenotypes in India

HBV genotypes A and D have been well documented from different parts of mainland India [49,61-66]. In two different studies from northern India, genotypes A and D were found to be equally prevalent [49,62]. However, another study from the same region reported genotype D to be predominant with a low frequency of genotype A in northern Indian HBV infected patients [65,66], which was comparable to the HBV genotype distribution documented from western and southern parts of India [52,61]. In sharp contrast to rest of the parts of India, the eastern part of India presents an interesting epidemiology of three different HBV genotypes (genotypes A, C and D) in comparable proportions [52,63,64].

**Figure 2:**
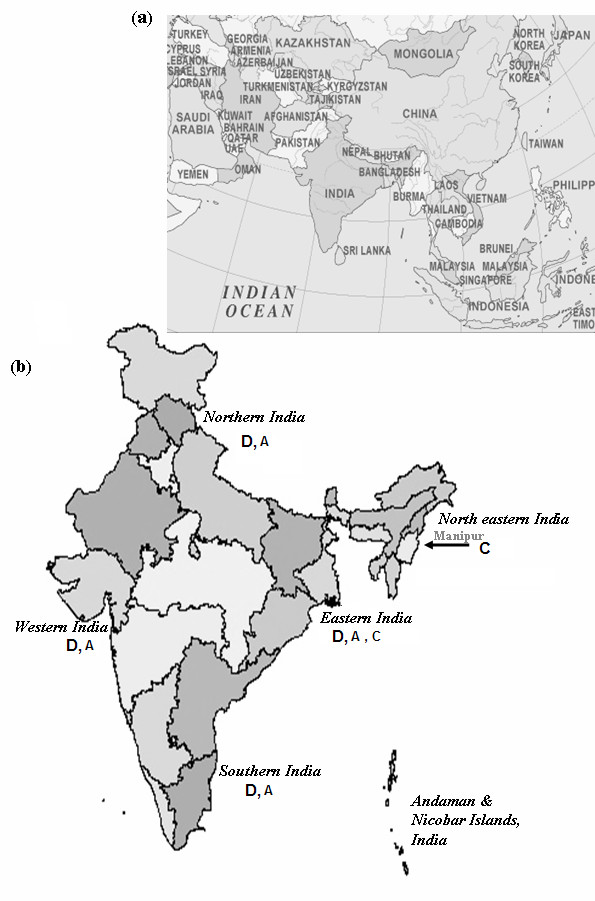
**India and its neighbouring countries**. (a) Geographical location of India with respect to neighbouring Asian countries. (b) Different parts of India and their HBV genotype distribution (denoted by alphabets A, C and D) are shown. The prevalent HBV genotype is denoted by bold alphabet.

Apart from only one study on subgenotypes of genotype A [67], there is a lack of data on the distribution of subgenotypes of HBV genotypes A and D in northern, western and southern parts of India. Most of the available information on the distribution of HBV subgenotypes of genotype A, C and D is available from eastern India only. In the eastern part of India, subgenotypes Aa/A1, Cs/C1, D1, D2, and D3 are prevalent [63,64]. In addition a novel subgenotype of D, designated as D5 was identified and characterized by complete genome sequencing of HBV isolates from Eastern India [64,15]. Based on the phylogenetic analysis and high nucleotide sequence similarity with south East Asian subgenotype Cs/C1 strain, genotype C strains from eastern Indian patients was suggested to be a recent introduction to eastern Indian population [52,63]. Thus, the eastern part of India is of great significance from the perspective of changing scenario of HBV epidemiology with presence of three distinct genotypes of HBV and four distinct subgenotypes of genotype D within a population with similar ethnic background. The HBV genotype distribution reported from differen parts of india has been shown in Figure 2.

On the other hand, in the Andaman and Nicobar islands, genotype D among three different primitive tribes (the Onge, the Andamanese, and the Nicobarese) was detected and its introduction from the people of mainland India was suggested [68]. In contrast, genotype C (subgenotype Cs/C1) was found exclusively among the Jarawas that was suggested to reflect their history of migration to the islands, long back [69].

## Prevalence of clinically important HBV mutants

An important aspect of the global HBV epidemiology is the emergence and increasing significance of HBeAg negative infections as well as the distribution and significance of HBV mutants, which are associated with suppression of the HBeAg synthesis and persistent infection. It is well known that the prevalence of e negative chronic hepatitis B and its molecular basis varies geographically with the prevalent HBV genotypes [70]. However, very little information on the prevalence and molecular epidemiology of HBeAg negative chronic infections is available from India. In Mediterranean populations, genotype D has been shown to present an extremely high prevalence of HBeAg negative chronic HBV infection, associated with HBV mutants in the PC region. Interestingly, despite the prevalence of genotype D in Northern and western parts of India, comparatively low prevalence of basal core promoter and precore mutant (33 – 37%) have been reported amongst HBeAg negative chronic HBV patients [71,72]. Moreover, PC mutation has not been found to be associated with severe liver disease, rather it was shown to favours the asymptomatic state in the western Indian population [71]. Although the prevalence of BCP mutations among Eastern Indian patients (32.5%) was similar to northern Indian patients (36%), but the prevalence of PC mutation in eastern India (18%) was found to be much lower in HBeAg negative CHB patients, compared to other parts of India [57,73].

## Incidence of HBV related HCC in India

Cancer is not a notifiable disease in India, and registration of incident cancer cases is done by means of active case finding. Although HCC cases are under reported in India, but association studies on the available cases indicate that chronic HBV infection is the most important factor responsible for the development of HCC, in India [74].

In a comparison based study of Indian Cancer Registries by Sen et al., [46], the incidence of HCC was found to be very much lower in comparison to the neighbouring countries of East Asia. An age standardized HCC incidence rate (ASR) of 5.3 and relative frequency of 4.8% was reported in males. However among the women, relative frequency of 3.1% and ASR of 3.9 was documented. Moreover, a recent study found the incidence of HCC in India to be low enough and excluded liver from the list of high-risk cancer site among Indians [75].

## Interesting molecular epidemiology of HBV in the Eastern part of India: scope of research

Thus, compared to other parts of India, the distribution patterns of HBV genotypes/subgenotypes and mutants is characteristically distinct in eastern part, where in addition to HBV genotypes A and D, genotype C is also present in a comparable proportion. This genotype is suggested as recently introduced and confined to this part of India. It was thus interesting to determine the routes of introduction of this south East Asian strain of HBV (Cs/C1), using molecular evolutionary techniques. The simultaneous presence of three different genotypes in the same population of eastern India is unique, providing opportunity to directly compare the clinical significance of HBV genotypes in disease manifestations and also in studying the importance of clinically relevant mutants in this population.

In the eastern part of India, despite high prevalence of chronic HBV infection and distribution of HBV subgenotype Aa/A1 and subgenotype Cs/C1, the incidence of HCC is notably low. This is in sharp contrast to HCC prevalence in sub-Saharan Africa and East Asian countries, where similar genotypes and subgenotypes (Aa/A1, Cs/C1) of HBV are prevalent. As the HBV *x *gene plays an important role in development of HCC, comparison of genetic variability of the HBV *x *gene region of Indian HBV genotype A and C isolates with isolates from sub Saharan Africa and East Asian might provide important clues. However, reports on genetic variability of HBx from other parts of the world are extremely rare, while no reports focusing the genetic variability of HBx from Indian HBV strains are available. It is also interesting to look for mutations of p53 gene, codon 249 in particular, which is significantly associated with AFB_1 _exposure and HBV related HCC cases in sub-Saharan Africa and East Asian countries. Apart from only one account documenting extremely low occurrence of p53 gene codon 249 mutation in northern Indian HCC patients [76], no reports are available on this aspect from the Indian subcontinent.

With the advent of sensitive amplification based assays, low quantities of HBV DNA have been frequently detected in the serum or liver or peripheral blood leukocytes (PBL) among HBsAg negative, antiHBc and/or antiHBs positive subjects (occult HBV infection). However, specific investigation on occult HBV DNA in the PBL and associated variants is extremely scanty. In a previous study from India, HBV DNA specifically with G145R immune escape mutation was shown to persist for long in the peripheral blood leukocytes (PBL) of HBV infected subjects [77]. Considering the importance of this observation in the HBV compartmentalization, transmission and epidemiology, studies focusing on the genetic variability of HBV DNA and its relevance in long persistence in the PBL was necessary.

Taking the advantage of the distribution of three distinct HBV genotypes within the same population of eastern India the thesis work was aimed to (i) study the molecular epidemiology and clinical significance of hepatitis B virus genotypes, core promoter and precore mutations in eastern India, (ii) identify possible routes of introduction of HBV genotype C in Eastern India, (iii) analyze the HBV *x *gene variability and its implications in Eastern Indian HBV carriers, (iv) determine the prevalence of the specific mutation of p53 gene (at codon 249), (v) study the transmission potentiality of HBV among family members of asymptomatic/inactive HBV carriers and (vi) study the genetic variability of HBV isolates persisting in the PBL.

## Summary of the experimental results obtained in the thesis

Geographically, eastern part of India is contiguous with the northeastern part of India, the later being physically and anthropologically attached to South East Asia. From this perspective, the appearance of HBV genotype C (prevalent HBV genotype in Southeast Asian countries) in north eastern and eastern India was well anticipated, and thus a different epidemiology of HBV genotypes in these regions was expected. The results of this thesis work, based on the analysis of different genetic regions (*surface, precore/core, x*) of the HBV genome clearly established the presence of three different HBV genotypes (genotypes A, C and D) in the eastern Indian population [78-80]. This unique distribution of three distinct genotypes in the eastern Indian population provided an opportunity to directly compare the clinical significance of three distinct genotypes in the same geo-ethnic population.

The comparison of clinical and virological characteristics between HBV genotypes A, C and D revealed the higher potentials of genotypes A and C in causing disease severity in this part of India, as they were associated with prolonged HBeAg positivity, higher ALT levels, higher viremia and, higher prevalence of mutations in the BCP region (at nucleotides 1762^T^/1764^A^) [79]. This study also indicated that precore mutation (1896^A^) does not have a prognostic role in predicting progress towards liver disease in this part of India [79]. More interestingly, infection with a particular HBV genotype was found to be associated with certain epidemiological risk factors; genotype D infection with history of jaundice in family or childhood or intrafamilial transmission, an important mode of transmission in this community, while percutaneous injury (frequent injection, needle prick, body piercing, use of unsterilized blade in community barber's shop), were associated with genotypes A and C infections [79].

To further elucidate the changing epidemiology of HBV infections and to explore the routes of introduction of HBV genotype C in this part of India, two different groups of subjects were studied. One of the groups included IDUs from the north eastern state of Manipur, who are well known to be exposed to the epidemics of intravenous drug abuse and parenteral viral infections from south East Asian countries, by virtue of sharing of drugs and injecting instruments. Another group examined included HBV infected subjects from a tribe (the Karen, considered a community in India) migrated from Myanmar. Both the study groups were selected based on the fact that they had well epidemiological links with both the south East Asian population and eastern Indian population. Analysis of the HBV genotypes among the IDUs in the present study well correlated with the hypothesis of spread of genotype C through drug routes [81]. On the other hand, as expected, genotype C was also found to be the prevalent strain of HBV among the Karen community of Andaman & Nicobar (A & N) islands, which well corroborated with the migration history of this community nearly 80 years ago, from the Southeast Asian country, Myanmar [82]. Nevertheless, considering the geographical separation of the A & N Islands, the possibility of spread of genotype C from the people of these islands (Karen) seems to be rather difficult. However, the northeastern states of India including Manipur are well connected to the eastern parts of India by various means and thus frequented by people from these states. Apart from HBV infection, the rapid change of epidemiology of HCV and HIV in the north eastern and eastern parts of India suggests the introduction of HBV genotype C through the northeastern states, through mobile and travelling population [81].

The thesis work was also unique, as it revealed for the first time, the genetic variability of the *x *gene region of the HBV strains circulating in the eastern part of India. Phylogenetic analysis of the *x *gene further confirmed the presence of HBV subgenotypes Aa/A1, Cs/C1, D1, D2, D3 and D5 in the serum of infected individuals [80]. The present study based on analysis of sequence and predicted structure of the HBx and its functional domains revealed the possible basis of genotype/subgenotype specific differences in the hepatocarcinogenic properties of HBV strains. It also suggested that the proline- serine rich hypervariable region (PSR) located in the N terminal part of HBx primarily determines most of the genotype/subgenotype specificity of the HBx [80]. During this study, detailed analysis of HBx sequences retrieved from the GenBank also demonstrated that certain hepatocarcinogenic mechanisms may act in a HBx genotype/subgenotype dependent [83]. It also revealed that frequent loss of HBx genetic region is a unique feature of HBV strains circulating in our population, and low genetic variability in the *x *gene region, compared to HBV strains from other countries. The occurrences of sporadic mutations, insertions, deletions or truncations previously reported to be prevalent in HCC patients from other countries was found to be extremely low, in the *x *gene region and changes specific for any particular clinical outcome were not observed in this study [80]. Apart from the low genetic variability of the HBx in the present study, the codon 249 mutation of p53 gene were not detected in any of the samples in the present study. Taken together, the data suggested a possible explanation for the low incidence of HBV-AFB_1 _related HCC in the population [80].

In the thesis, attempts were also made to investigate the transmission patterns in the families of incidentally detected HBsAg carriers or individuals with occult HBV infection, through intrafamilial modes. The results indicated that the clinical status of the index case does not influence the aggregation pattern of intrafamilial infection in this population. Although the percentage was small, the present study, based on advanced molecular evolutionary analyses, confirmed for the first time that occult HBV infection could indeed be transmissible through apparent non-sexual, non-parenteral contacts in a familial setting [78]. It also revealed that sexual transmission was not the predominant mode of transmission in some families, even when one of spouses had high levels of viremia, suggesting that sexual transmission in adult life may not be an efficient mode of transmission in this population [78]. In this study, genotype D was found to be prevalent among the HBsAg positive index cases while genotype A was prevalent among the HBsAg negative (occult HBV infection) family members, that supported the different epidemiology of HBV genotypes, even in a familial setting [78].

Finally, the present thesis work was archetype in examining the genetic variability of HBV in the peripheral blood leukocytes (PBL). Attempts to characterize the HBV sequences revealed the compartment restricted predominance of HBV genotype A (subgenotype Ae/A2) specific sequences with a potent immune escape G145R mutation in the PBL of majority of the study subjects from this study population. Interestingly, entirely different HBV genotypes/subgenotypes (C, D or subgenotype Aa/A1) were found to predominate in the sera of the same study population. The highly contrasting prevalence of subgenotype Ae/A2 associated with the immune escape G145R mutation in serum and PBL suggested that the HBV DNA and expressed viral antigen in the serum, liver and PBL are under different selection pressure. During this work, detailed comparison and analysis of the pregenomic RNA base pairing of different HBV genotypes also suggested a potential molecular mechanism that explained the specific selection of the G145R mutation in the context of subgenotype Ae/A2 specific sequences and higher immune selection on the PBL [Datta et al. Manuscript under review].

## Conclusion

In conclusion, the results of this thesis sheds light on many important aspects of HBV molecular epidemiology that are very much important for identifying the population at risk of acquiring HBV and developing severe disease, and also pose a risk of transmission through different modes. This information are essential for determining the risk factors associated with HBV infections, to formulate necessary preventive measures to lessen the burden of new infections and spread of newly introduced genotype to other parts, from eastern India. Moreover, considering the higher pathogenic potentials associated with certain HBV genotypes, the results of the thesis work will be helpful in prognosis and better management of HBV infected subjects.

Emergence of new genotypes/subgenotypes, clinically important mutations have immense importance in determining the clinical outcome, efficacy of vaccination and therefore strict surveillance of these variants are extremely important. Hope that the present study will advance the understanding of changing molecular epidemiology of HBV, and will also help in formulation of effective preventive measures. Last but most important, the results of this thesis work, demonstrating compartment specific high prevalence of HBV DNA associated with vaccine/immune escape mutation in the PBL of HBsAg negative subjects have extremely important implications in the field of transfusion medicine, organ transplantation, and in vaccination strategies, and thus need further investigations. Finally, together with contributing unique data on molecular epidemiology of HBV in India, this thesis work also open new avenues for further studying the molecular virology of HBV.

## Competing interests

The author declares that they have no competing interests.

## Biographical summary of the author

The author studied Zoology at the Bachelor and Master Degree level and later joined the Indian Council of Medical Research Virus Unit, for doctoral research. He specializes in the fields of molecular diagnostics, epidemiology, evolution and genomics of viruses.

## Authors' contributions

The author compiled the above information for writing the background section of his doctoral thesis entitled "*Molecular Epidemiology of Hepatitis B virus in Eastern India: Role of Genotypes, X gene Variability and Disease Outcome*", which has been approved by the doctoral committee of Jadavpur University, for the award of PhD degree.

## References

[B1] Hepatitis B Fact sheets WHO. http://www.who.int/mediacentre/factsheets/fs204/en.

[B2] Lavanchy D (2004). Hepatitis B virus epidemiology, disease burden, treatment, and current and emerging prevention and control measures: a review. J Viral Hepat.

[B3] Ganem D, Schneider RJ, Knipe DM, Howley PM (2001). Hepadnaviridae: the viruses and their replication. Fields Virology.

[B4] Seeger C, Mason WS (2000). Hepatitis B Virus Biology. Microbiol Mol Biol Rev.

[B5] Mizokami M, Orito E, Ohba K, Ikeo K, Lau JY, Gojobori T (1997). Constrained evolution with respect to gene overlap of hepatitis B virus. J Mol Evol.

[B6] Kidd-Ljunggren K, Miyakawa Y, Kidd AH (2002). Genetic variability in hepatitis B viruses. J Gen Virol.

[B7] Bonhoeffer S, Sniegowski P (2002). Virus evolution: the importance of being erroneous. Nature.

[B8] Okamoto H, Imai M, Kametani M, Nakamura T, Mayumi M (1987). Genomic heterogeneity of hepatitis B virus in a 54-year-old woman who contracted the infection through materno-fetal transmission. Jpn J Exp Med.

[B9] Orito E, Mizokami M, Ina Y, Moriyama EN, Kameshima N, Yamamoto M, Gojobori T (1989). Host-independent evolution and a genetic classification of the hepadnavirus family based on nucleotide sequences. Proc Natl Acad Sci USA.

[B10] Sterneck M, Gunther S, Gerlach J, Naoumov NV, Santantonio T, Fischer L, Rogiers X, Greten H, Williams R, Will H (1997). Hepatitis B virus sequence changes evolving in liver transplant recipients with fulminant hepatitis. J Hepatol.

[B11] Blackberg J, Kidd-Ljunggren K (2000). Occult hepatitis B virus after acute self-limited infection persisting for 30 years without sequence variation. J Hepatol.

[B12] Hannoun C, Horal P, Lindh M (2000). Long-term mutation rates in the hepatitis B virus genome. J Gen Virol.

[B13] Osiowy C (2006). Detection of HBsAg Mutants. J Med Virol.

[B14] Schaefer S (2005). Hepatitis B virus: Significance of genotypes. J Viral Hepat.

[B15] Schaefer S (2007). Hepatitis B virus taxonomy and hepatitis B virus genotypes. World J Gastroenterol.

[B16] Okamoto H, Tsuda F, Sakugawa H, Sastrosoewignjo RI, Imai M, Miyakawa Y, Mayumi M (1988). Typing hepatitis B virus by homology in nucleotide sequence: comparison of surface antigen subtypes. J Gen Virol.

[B17] Norder H, Courouce AM, Magnius LO (1994). Complete genomes, phylogenetic relatedness, and structural proteins of six strains of the hepatitis B virus, four of which represent two new genotypes. Virology.

[B18] Stuyver L, De Gendt S, van Geyt C, Zoulim F, Fried M, Schinazi RF, Rossau R (2000). A new genotype of hepatitis B virus: Complete genome and phylogenetic relatedness. J Gen Virol.

[B19] Arauz-Ruiz P, Norder H, Robertson BH, Magnius LO (2002). Genotype H: A new Amerindian genotype of hepatitis B virus revealed in Central America. J Gen Virol.

[B20] Echevarria JM, Avellon A (2006). Hepatitis B virus genetic diversity. J Med Virol.

[B21] Kuang SY, Jackson PE, Wang JB, Lu PX, Munoz A, Qian GS, Kensler TW, Groopman JD (2004). Specific mutations of hepatitis B virus in plasma predict liver cancer development. Proc Natl Acad Sci USA.

[B22] Chen WN, Oon CJ, Lim GK (2001). Frequent occurrence of hepatitis B virus surface antigen mutants in subtype adw in vaccinated Singapore infants. Vaccine.

[B23] Tanaka Y, Mizokami M (2007). Genetic Diversity of Hepatitis B Virus as an important factor associated with differences in clinical outcomes. J Infect Dis.

[B24] Kato H, Orito E, Sugauchi F, Ueda R, Gish RG, Usuda S, Miyakawa Y, Mizokami M (2001). Determination of hepatitis B virus genotype G by polymerase chain reaction with hemi-nested primers. J Virol Methods.

[B25] Vieth S, Manegold C, Drosten C, Nippraschk T, Gunther S (2002). Sequence and phylogenetic analysis of hepatitis B virus genotype G isolated in Germany. Virus Genes.

[B26] Hallett RL, Ngui SL, Meigh RE, Mutton KJ, Boxall EH, Teo CG (2004). Widespread dissemination in England of a stable and persistent hepatitis B virus variant. Clin Infect Dis.

[B27] Koppelman MH, Zaaijer HL (2004). Diversity and origin of hepatitis B virus in Dutch blood donors. J Med Virol.

[B28] Parkin DM, Bray F, Ferlay J, Pisani P (2005). Global cancer statistics, 2002. CA Cancer J Clin.

[B29] Hussain SP, Schwank J, Staib F, Wang XW, Harris CC (2007). TP53 mutations and hepatocellular carcinoma: insights into the etiology and pathogenesis of liver cancer. Oncogene.

[B30] Montesano P, Hainaut P, Wild CP (1997). Hepatocellular Carcinoma: From Gene to Public Health. J Natl Cancer Inst.

[B31] Stern MC, Umbach DM, Yu MC, London SJ, Zhang ZQ, Taylor JA (2001). Hepatitis B, Aflatoxin B1, and p53 Codon 249 mutation in hepatocellular carcinomas from Guangxi, People's Republic of China, and a meta-analysis of existing studies. Cancer Epidemiol Biomarkers Prev.

[B32] Wang XW, Forrester K, Yeh H, Feitelson MA, Gu JR, Harris CC (1994). Hepatitis B virus X protein inhibits p53 sequence-specific DNA binding, transcriptional activity, and association with transcription factor ERCC3. Proc Natl Acad Sci USA.

[B33] Truant R, Antunovic J, Greenblatt J, Prives C, Cromlish JA (1995). Direct interaction of the hepatitis B virus HBx protein with p53 response element-directed transactivation. J Virol.

[B34] Groisman IJ, Fotouhi-Ardakani N, Schecter RL, Woo A, Alaoui-Jamali MA, Batist G (2000). Modulation of Glutathione S-Transferase Alpha by Hepatitis B Virus and the Chemopreventive Drug Oltipraz. J Biol Chem.

[B35] Madden CR, Finegold MJ, Slagle BL (2002). Altered DNA Mutation Spectrum in Aflatoxin B1-Treated Transgenic Mice That Express the Hepatitis B Virus X Protein. J Virol.

[B36] Smela ME, Currier SS, Bailey EA, Essigmann JM (2001). The chemistry and biology of Aflatoxin B1: from mutational spectrometry to carcinogenesis. Carcinogenesis.

[B37] Bouchard MJ, Schneider RJ (2004). The Enigmatic X Gene of Hepatitis B Virus. J Virol.

[B38] Kidd-Ljunggren K, Oberg M, Kidd AH (1995). The hepatitis B virus X gene: analysis of functional domain variation and gene phylogeny using multiple sequences. J Gen Virol.

[B39] Zaidi AKM, Awasthi S, deSilva HJ (2004). Burden of infectious diseases in South Asia. Brit Med J.

[B40] Gupta S, Singh S (2006). Hepatitis B and C virus co-infections in human immunodeficiency virus positive North Indian patients. World J Gastroenterol.

[B41] Bhattacharya P, Chandra PK, Datta S, Banerjee A, Chakraborty S, Rajendran K, Basu SK, Bhattacharya SK, Chakravarty R (2007). Significant increase in HBV, HCV, HIV and syphilis infections among blood donors in West Bengal, Eastern India 2004–2005: Exploratory screening reveals high frequency of occult HBV infection. World J Gastroenterol.

[B42] (2002). Prevention of Hepatitis B in India- An Overview.

[B43] Murhekar MV, Murhekar KM, Das D, Arankalle VA, Sehgal SC (2000). Prevalence of hepatitis B infection among the primitive tribes of Andaman and Nicobar islands. Indian J Med Res.

[B44] Murhekar MV, Murhekar KM, Sehgal SC (2004). Age-specific prevalence of hepatitis B infection among the Karen in the Andaman and Nicobar Islands, India. Trop Doct.

[B45] Murhekar MV, Murhekar KM, Sehgal SC (2003). Alarming prevalence of hepatitis B among the Jarawas- a primitive Negrito tribe of Andaman and Nicobar Islands, India. J Viral Hepatitis.

[B46] Sen U, Sankaranarayanan R, Mandal S, Ramanakumar AV, Parkin DM, Siddiqi M (2002). Cancer patterns in eastern India: The first report of the Kolkata Cancer registry. Int J Cancer.

[B47] Basu A, Mukherjee N, Roy S, Sengupta S, Banerjee S, Chakraborty M, Dey B, Roy M, Roy B, Bhattacharyya NP, Roychoudhury S, Majumder PP (2003). Ethnic India: A genomic view, with special reference to peopling and sructure. Genome Res.

[B48] Sahoo S, Singh A, Himabindu G, Banejee J, Sitalaximi T, Gaikwad S, Trivedi R, Endicott P, Kivisild T, Metspalu M, Villems R, Kashyap VK (2006). A prehistory of Indian Y Chromosome: Evaluating demic diffusion scenarions. Proc Natl Acad Sci USA.

[B49] Thakur V, Guptan RC, Kazim SN, Malhotra V, Sarin SK (2002). Profile, spectrum and significance of HBV genotypes in chronic liver disease patients in the Indian subcontinent. J Gastroenterol Hepatol.

[B50] Bamshad M, Kivisild T, Watkins WS, Dixon ME, Ricker CE, Rao BB, Naidu M, Prasad BVR, Reddy PG, Rasanayagam A, Papiha SS, Villems R, Redd AJ, Hammer MF, Nguyen SV, Carroll ML, Batzer MA, Jorde LB (2001). Genetic evidence on the origins of Indian caste populations. Genome Res.

[B51] Beyrer C, Razak MH, Lisam K, Chen J, Lui W, Yu XF (2000). Overland heroin trafficking routes and HIV-1 spread in south and south-east Asia. AIDS.

[B52] Vivekanandan P, Abraham P, Sridharan G, Chandy G, Raghuraman S, Daniel H, Subramaniam T (2004). Distribution of Hepatitis B Virus Genotypes in Blood Donors and Chronically Infected Patients in a Tertiary Care Hospital in Southern India. Clin Infect Dis.

[B53] Chaudhuri S, Das S, Chowdhury A, Santra A, Bhattacharya SK, Naik TN (2005). Molecular epidemiology of HCV infection among acute and chronic liver disease patients in Kolkata, India. J Clin Virol.

[B54] Nayak NC, Panda SK, Bhan MK, Guha DK, Zuckerman AJ (1987). Dynamics and impact of perinatal transmission of hepatitis B virus in North India. J Med Virol.

[B55] Chakravarty R, Chowdhury A, Chaudhuri S, Santra A, Neogi M, Raendran K, Panda CK, Chakravarty M (2005). Hepatitis B infection in Eastern Indian families: Need for screening of adult siblings and mothers of adult index cases. Public Health.

[B56] Banerjee A, Chakravarty R, Mondal PN, Chakraborty MS (2005). Hepatitis B virus genotype D infection among antenatal patients attending a maternity hospital in Calcutta, India: assessment of infectivity status. Southeast Asian J Trop Med Public Health.

[B57] Chowdhury A, Santra A, Chakravorty R, Banerji A, Pal S, Dhali GK, Datta S, Banerji S, Manna B, Chowdhury SR, Bhattacharya SK, Mazumder DG (2005). Community-based epidemiology of hepatitis B virus infection in West Bengal, India: prevalence of hepatitis B e antigen-negative infection and associated viral variants. J Gastroenterol Hepatol.

[B58] Petersen NJ, Barrett DH, Bond WW, Berquist KR, Favero MS, Bender TR, Maynard JE (1976). Hepatitis B surface antigen in saliva, impetiginous lesions, and the environment in two remote Alaskan villages. Appl Environ Microbiol.

[B59] Maddrey WC (2000). Hepatitis B: an important public health issue. J Med Virol.

[B60] Thakur V, Kazim SN, Guptan RC, Malhotra V, Sarin SK (2003). Molecular epidemiology and transmission of hepatitis B virus in close family contacts of HBV-related chronic liver disease patients. J Med Virol.

[B61] Gandhe SS, Chadha MS, Arankalle VA (2003). Hepatitis B virus genotypes and serotypes in Western India: Lack of clinical significance. J Med Virol.

[B62] Kumar A, Kumar SI, Pandey R, Naik S, Aggarwal R (2005). Hepatitis B virus genotype A is more often associated with severe liver disease in northern India than is genotype D. Indian J Gastroenterol.

[B63] Banerjee A, Datta S, Chandra PK, Roychowdhury S, Panda CK, Chakravarty R (2006). Distribution of hepatitis B virus genotypes: Phylogenetic analysis and virological characteristics of Genotype C circulating among HBV carriers in Kolkata, Eastern India. World J Gastroenterol.

[B64] Banerjee A, Kurbanov F, Datta S, Chandra PK, Tanaka Y, Mizokami M, Chakravarty R (2006). Phylogenetic relatedness and genetic diversity of hepatitis B virus isolates in Eastern India. J Med Virol.

[B65] Chattopadhyay S, Das BC, Hussain Z, Kar P (2006). Hepatitis B virus genotypes in acute and fulminant hepatitis patients from north India using two different molecular genotyping approaches. Hepatol Res.

[B66] Chattopadhyay S, Das BC, Kar P (2006). Hepatitis B virus genotypes in chronic liver disease patients from New Delhi, India. World J Gastroenterol.

[B67] Tanaka Y, Hasegawa I, Kato T, Orito E, Hirashima N, Acharya SK, Gish RG, Kramvis A, Kew MC, Yoshihara N, Shrestha SM, Khan M, Miyakawa Y, Mizokami M (2004). A case control study for differences among hepatitis B virus infections of genotypes A (subtypes Aa and Ae) and D. Hepatology.

[B68] Arankalle VA, Murhekar KM, Gandhe SS, Ramdasi AY, Padbidri, Sehgal SC (2003). Hepatitis B virus: predominance of genotype D in primitive tribes of the Andaman and Nicobar Islands, India (1989–1999). J Gen Virol.

[B69] Murhekar MV, Chakraborty R, Murhekar KM, Banerjee A, Sehgal SC (2006). Hepatitis B virus genotypes among the Jarawas: A primitive Negrito tribe of Andaman and Nicobar Islands, India. Arch Virol.

[B70] Kramvis A, Kew M (2005). Relationship of genotypes of hepatitis B virus to mutations, disease progression and response to antiviral therapy. J Viral Hepat.

[B71] Gandhe SS, Chadha MS, Walimbe AM, Arankalle VA (2003). Hepatitis B virus: prevalence of precore/core promoter mutants in different clinical categories of Indian patients. J Viral Hepat.

[B72] Chauhan R, Kazim SN, Bhattacharjee J, Sakhuja P, Sarin SK (2006). Basal Core Promoter, Precore Region Mutations of HBV and Their Association With e Antigen, Genotype, and Severity of Liver Disease in Patients With Chronic Hepatitis B in India. J Med Virol.

[B73] Banerjee A, Banerjee S, Chowdhury A, Santra A, Chowdhury S, Roychowdhury S, Panda CK, Bhattacharya SK, Chakravarty R (2005). Nucleic acid sequence analysis of BCP/precore/core region of hepatitis B virus isolated from chronic carriers of the virus from Kolkata, Eastern India: low frequency of mutation in the precore region. Intervirology.

[B74] Raza SA, Clifford GM, Franceschi S (2007). Worldwide variation in the relative importance of hepatitis B and hepatitis C viruses in hepatocellular carcinoma: a systematic review. Brit J Cancer.

[B75] Nandakumar A, Gupta PC, Gangadharan P, Visweswara RN, Parkin DM (2005). Geography pathology revisited: Development of an atlas of cancer in India. Int J Cancer.

[B76] Katiyar S, Dash BC, Thakur V, Guptan RC, Sarin SK, Das BC (2000). P53 tumor suppressor gene mutations in hepatocellular carcinoma patients in India. Cancer.

[B77] Chakravarty R, Neogi M, Roychowdhury S, Panda CK (2002). Presence of hepatitis B surface antigen mutant G145R DNA in the peripheral blood leukocytes of the family members of an asymptomatic carrier and evidence of its horizontal transmission. Virus Res.

[B78] Datta S, Banerjee A, Chandra PK, Chowdhury A, Chakravarty R (2006). Genotype, phylogenetic analysis, and transmission pattern of occult hepatitis B virus (HBV) infection in families of asymptomatic HBsAg carriers. J Med Virol.

[B79] Datta S, Biswas A, Chandra PK, Banerjee A, Panigrahi R, Mahapatra PK, Chakrabarti S, Panda CK, Chakravarty R (2008). Molecular epidemiology and clinical significance of hepatitis B virus genotypes, core promoter and precore mutations in Eastern India. Intervirology.

[B80] Datta S, Banerjee A, Chandra PK, Biswas A, Panigrahi R, Mahapatra PK, Panda CK, Chakrabarti S, Bhattacharya SK, Chakravarty R (2008). Analysis of hepatitis B virus X gene phylogeny, genetic variability and its impact on pathogenesis: implications in Eastern Indian HBV carriers. Virology.

[B81] Datta S, Banerjee A, Chandra PK, Mahapatra PK, Chakrabarti S, Chakravarty R (2006). Drug trafficking routes and hepatitis B in injection drug users, Manipur, India. Emerg Infect Dis.

[B82] Datta S, Chandra PK, Banerjee A, Chakravarty R, Murhekar KM, Murhekar MV (2007). Predominance of Hepatitis B virus genotype C among Karens, the 'old settlers' of Andaman and Nicobar Islands, India. Arch Virol.

[B83] Datta S, Banerjee A, Chandra PK, Chakravarty R (2007). Pin1-HBx Interaction: A step towards understanding the significance of HBV genotypes in hepatocarcinogenesis. Gastroenterology.

